# The Clinical Relevance of the Rectosigmoid Brake in Surgical Disorders and Therapies: A Systematic Review of Colonic Manometry Studies

**DOI:** 10.1111/nmo.70288

**Published:** 2026-03-19

**Authors:** James A. Penfold, Cameron I. Wells, Ian P. Bissett, Gregory O'Grady

**Affiliations:** ^1^ Department of Surgery, Faculty of Medical and Health Sciences The University of Auckland Auckland New Zealand; ^2^ Department of Surgery Auckland District Health Board Auckland New Zealand; ^3^ Auckland Bioengineering Institute The University of Auckland Auckland New Zealand

**Keywords:** colonic motility, fecal incontinence, ileus, low anterior resection syndrome, sacral nerve stimulation

## Abstract

**Background:**

The rectosigmoid brake (RSB) regulates rectal filling via retrograde cyclic motor patterns in the distal colon. Disruption has been linked to postoperative ileus (POI), low anterior resection syndrome (LARS), fecal incontinence (FI), and acute colonic pseudo‐obstruction (ACPO). We synthesized manometric evidence to clarify the RSB's clinical relevance.

**Methods:**

A systematic search of Ovid MEDLINE and Embase (March 2025) for studies assessing distal colonic motility in adults with surgical or functional colorectal conditions. Thirty‐four studies met the inclusion criteria, including nine using high‐resolution manometry. Data were qualitatively synthesized by condition.

**Key Results:**

Altered RSB activity was a consistent finding. In POI (7 studies; 2 HRM), the HRM studies showed immediate postoperative hyperactive distal cyclic activity (~2–4 cycles/min) correlating with delayed return of bowel function, contradicting older low‐resolution reports of quiescence. In LARS (4 studies; 3 HRM), post‐prandial cyclic patterns and propagating sequences were blunted after rectosigmoid resection. In FI (5 studies; 2 HRM), the brake was suppressed; sacral nerve stimulation increased distal retrograde contractions with parallel symptom gains. In ACPO (1 HRM case study), recordings showed disordered, non‐propagating hyperactive activity consistent with distal functional obstruction rather than hypoactivity.

**Conclusions:**

Across POI, LARS, FI, and ACPO, the RSB appears to be a unifying physiological mechanism and a promising physiological biomarker with diagnostic and therapeutic potential. Priorities include standardizing manometric definitions, establishing normative reference metrics, and advancing non‐invasive assessment (e.g., body‐surface colonic mapping) to enable RSB‐guided care and translation to practice.

## Key Points

- The distal colon has a natural “brake” (the rectosigmoid brake) that controls how quickly stool enters the rectum, helping prevent urgency and maintain continence.
- When this brake misfires, different problems can result: after colorectal surgery it may become overactive and delay recovery; when weakened or removed it can lead to urgency or incontinence.
- This brake is a promising, measurable sign to guide care: it can be boosted by sacral nerve stimulation, and new non‐invasive tests may help doctors track it to predict recovery and tailor treatment.

## Graphical abstract

Colonic motility mapping reveals a meal‐induced rectosigmoid brake in healthy individuals that is disrupted, disorganized, or exaggerated following surgery and in motility disorders. These alterations underlie conditions such as ileus, pseudo‐obstruction, and fecal incontinence, and may be modulated by sacral neuromodulation.

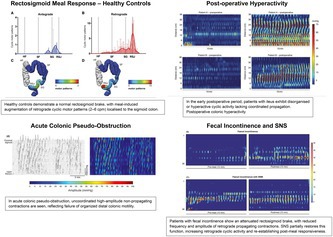

## 1 Introduction

Colonic motility is a widely studied but complex and multifactorial phenomenon, underpinning normal bowel function and contributing to a spectrum of functional disorders when disrupted [1]. Disordered motility not only manifests in chronic conditions such as constipation, diarrhea, and fecal incontinence (FI) but also plays a central role in the recovery and complications that follow abdominal surgery [2, 3, 4, 5]. These clinical consequences highlight the importance of understanding regulatory mechanisms within the distal colon.

Historically, O'Beirne postulated a rectosigmoid sphincter in the early 19th century, and although a discrete anatomical structure is not supported by modern evidence, physiological high‐volume activity in this region has since been confirmed [6]. The “colonic gatekeeper” was described by Rao and Welcher et al. who found an intrinsic braking mechanism in the rectum that alters the flow of colonic contents [7]. More recent research refined this concept as the ‘rectosigmoid brake’ (RSB), whereby retrograde cyclic motor patterns regulate rectal filling and promote coordinated defecatory activity [2, 3, 8, 9, 10]. In healthy individuals, the RSB is characterized by predominantly retrograde propagating sequences occurring at 2–6 cycles per minute, often triggered by rectal filling or postprandial stimuli [2, 8, 10, 11]. Lin et al. provided the first robust description of this mechanism, demonstrating retrograde cyclic motor patterns originating in the sigmoid colon and rectum that restrict antegrade transit and prevent premature rectal loading, thereby promoting coordinated bowel activity [1, 8].

Over the last decade, our understanding of colonic motility has advanced substantially. However, postoperative complications such as postoperative ileus (POI) and low anterior resection syndrome (LARS), along with disease states such as FI, continue to pose significant clinical challenges. Dysfunction of the RSB has been implicated in several surgical syndromes, including LARS, POI, and FI, where disrupted motility patterns and impaired RSB function are observed [2, 4, 5, 10]. Short‐term impacts of altered colonic motility postoperatively manifest as prolonged gastrointestinal (GI) transit resulting in postoperative ileus (POI) [12, 13]. Long‐term impacts of disrupting colonic motility are seen in patients with low anterior resection syndrome (LARS), which is hypothesized to be the result of loss of the RSB cyclic motor patterns [2]. These conditions impose considerable healthcare costs and substantially impair patient quality of life, prolong hospitalization, and affect recovery outcomes [14, 15, 16, 17, 18]. Understanding the influence of surgical interventions on the RSB and its role in POI or altered motility is critical for developing targeted therapies and improving patient outcomes.

Colonic manometry, particularly high‐resolution manometry (HRM), has facilitated the in‐depth characterization of motor patterns in both healthy and postoperative populations. These technologies have enabled the visualization and characterization of the RSB, contributing valuable insights into its function and dysfunction [1, 8]. Despite these advances, the current evidence base is fragmented. Studies are heterogeneous in design and patient populations, and definitions of motility events and normative data on rectosigmoid function are lacking. Moreover, while altered RSB activity has been observed across multiple surgical and pathological contexts, the mechanistic links to symptom generation and recovery remain poorly understood. These gaps limit the ability to draw firm conclusions about the physiological role of the RSB and its contribution to postoperative dysfunction, underscoring the need for a structured synthesis of the available literature.

This systematic review, therefore, aimed to comprehensively evaluate the literature on colonic motility investigated using colonic manometry, focusing on those studies using high‐resolution techniques. The primary objective was to synthesize existing evidence from colonic manometry studies that assess motility patterns in the distal colon, assessing the surgical relevance of the RSB and its potential contribution to postoperative dysmotility syndromes [9]. This review also sought to clarify the RSB's implications for postoperative care and therapeutic targeting by assessing its physiological role, dysfunction prevalence, and association with clinical outcomes. The findings may also guide the development of motility‐based biomarkers and targeted interventions such as neuromodulation to mitigate surgical bowel dysfunction. Ultimately, this work intended to establish a deeper understanding of the RSB's role in gastrointestinal (GI) physiology and its broader implications for surgical practice.

## 2 Methods

This review followed the guidelines outlined in the 2020 preferred guideline for Systematic Review Synthesis without Meta‐analysis (SWiM) [19]. The review protocol was registered prospectively in the PROSPERO database before initiating the literature search and study procedures (Registration ID: 1053180). Formal ethical approval was not required.

### 2.1 Search Strategy

A comprehensive literature search was carried out using Ovid MEDLINE and Embase. The search strategy targeted the title, abstract, and keyword fields using the terms “manometry” AND “colon* OR pancolon*” on March 24, 2025. These terms were linked using Boolean operators, and the search was unrestricted by date or geography but limited to English full texts.

### 2.2 Screening & Data Extraction

All search results were exported from the databases, and duplicates were removed following the methodology outlined by Bramer et al. [20]. Two reviewers (J.P. and C.W.) independently screened the titles and abstracts using the Rayyan.ai web‐based platform.

### 2.3 Inclusion and Exclusion Criteria

Included studies were those looking at adult humans (≥ 18 years) undergoing colonic manometry with surgically relevant conditions involving the colon or rectum. Manometry was required to be colonic in nature, i.e., with sensor coverage extending at least into the sigmoid colon (e.g., high‐resolution manometry, barometry, water‐perfused, or solid‐state systems) and analysis to identify colonic or specifically rectosigmoid motor patterns. Studies were excluded if they included only pediatric patients or studied anorectal manometry or balloon expulsion alone. Studies investigating constipation, including slow‐transit constipation, were excluded, as the review focused on predominantly surgical disorders; although constipation may rarely require colectomy, it was not considered a primary surgical condition. Only papers published in English were included. Publications excluded from consideration were conference abstracts, study protocols, qualitative studies, surveys, review articles, letters to the editor, and editorials.

### 2.4 Data Synthesis

All full texts were reviewed by one reviewer (JP), and a narrative synthesis approach was applied. Findings were extracted and grouped by disease state or surgical context to allow comparison across conditions. In addition, the quality and resolution of manometry data were assessed, with particular attention to differences between high‐resolution and low‐resolution techniques, given their implications for the accuracy and interpretability of motor pattern characterization. This approach was selected due to heterogeneity in study designs, populations, and outcome measures, which precluded formal meta‐analysis.

## 3 Results

A total of 1860 records were identified through the systematic search. After removal of duplicates and screening, 59 full texts were reviewed, of which 34 studies met the inclusion criteria (PRISMA Diagram, Figure S1). Of the 34 included studies, ten employed high‐resolution fiber‐optic systems, while the remaining 24 used conventional low‐resolution techniques (e.g., water‐perfusion, strain gauge, or barostat). Table 1 outlines conditions studied and techniques used.

**TABLE 1:** Categorical classification of studies and associated manometric resolution.

Category	Low resolution			High resolution	
	Water‐perfused	Strain gauge	Barostat	Fiber‐optic	Solid state
ACPO				[11]	
Colostomy	[21]				
Diverticulosis	[22, 23, 24, 25, 26, 27, 28]	[29]		[30]	
FI	[31, 32]	[33]		[10, 34]	
Ileus	[35, 36, 37, 38]	[39]		[4, 40]	
Inflammatory bowel disease	[41]				
LARS	[42]			[2, 3]	[43]
Medication	[44, 45, 46]	[47]	[48]	[49]	

Understanding colonic motility relies heavily on specialized measurement techniques. Manometry, which records intraluminal pressure via catheters with pressure sensors, has been the principal method used. Most studies employed low‐resolution manometry, with only nine utilizing fiber‐optic or solid state high‐resolution manometry (HRM). Some studies instead used water perfusion, barostat systems, or water perfusion catheters. HRM provides pressure measurements at closely spaced intervals (1–2.5 cm), yielding detailed spatiotemporal maps of colonic activity [50]. In contrast, low‐resolution catheters use fewer sensors spaced further apart, limiting recognition of colonic propagating sequences [51]. Specifically, low‐resolution studies must be interpreted cautiously, as their limited spatiotemporal detail both restricts accurate interpretation and contributes to heterogeneous findings. These shortcomings underscore the value of HRM, which has been demonstrated to offer markedly improved resolution for identifying colonic motility patterns [51, 52]. A large proportion of studies relied on low‐resolution techniques, making data interpretation more challenging. Reported metrics included cyclic motor patterns (CMPs), high‐amplitude propagating sequences (HAPS), and motility indices that quantify overall contractile activity [21, 39, 45, 47, 53, 54]. These results will discuss this evidence as it pertains to specific surgically relevant conditions.

### 3.1 Post Operative Ileus

Seven studies investigated postoperative colonic motility in postoperative ileus (POI), two of which used HRM [4, 35, 36, 37, 38, 39, 40] (Table 2). These HRM studies indicate that colonic electrical and motor activity does not routinely cease after surgery. Instead, both reported markedly hyperactive rectosigmoid function, with peak frequencies of 2–4 cycles per minute in the sigmoid colon [4]. Compared with non‐operative controls, the postoperative cyclic activity was substantially more pronounced, even compared to the normal fed state. These hyperactive cyclic motor patterns in the distal colon in the early postoperative setting oppose traditional assumptions of colonic atony and distension [4, 5, 36, 40]. Wells et al. investigated these in the first study to use HRM to measure postoperative colonic motility for an extended period, being more than 16 h after surgery [40]. Hyperactive CMPs were found to temporally correlate with symptoms of both abdominal distension and nausea [40]. Additionally, these data demonstrated that no patients experienced return of bowel function until after this hyperactive CMP activity had returned to near preoperative levels. Despite small sample sizes (*n* = 24), findings consistently indicate a hyperactive RSB, contributing to delayed transit in and representing a possible underlying mechanism of POI [55, 56].

**TABLE 2:** High resolution studies investigating post‐operative ileus (POI).

Author	Technique	Participants	Main findings	Limitations
Vather—2018	36‐channel HR fiber‐optic manometry, 1 cm sensor spacing, in distal colon	Eight patients undergoing right hemi‐colectomy and nine healthy controls	Hyperactive motility in all postoperative patients. Cyclic motor patterns increased in intensity and extent becoming nearly continuous (94%) with peak frequencies at 2–4 cpm in the sigmoid colon	On average only 1001 min (947–1278) of recording postoperatively
Wells—2023	36‐channel HR fiber‐optic manometry, 1 cm sensor spacing, in distal colon	Seven patients undergoing elective right hemi‐colectomy	Hyperactive cyclic motor patterns begun intra‐operatively, peaking in first 12 h postoperatively. These occupied 81.8% of recordings and gradually returned to normal over 4 days. High amplitude propagating sequences were absent in early recordings and their return temporally correlated with stool passage	Small sample size (*n* = 7). Impact of postoperative analgesia/meals not investigated

Together, these findings suggest that the RSB is not only a marker of colonic function but also a possible direct therapeutic target. In support of these data, one water‐perfusion study by Huge et al. demonstrated that the early postoperative motility in the colon exhibits ‘increased tone’ rather than ‘atony’ [36].

By contrast, two historical low‐resolution studies supported the opposite concept that postoperative colonic activity is characterized by quiescence, finding that left‐sided colonic surgery with an anastomosis was associated with a decrease in motility index [39]. These results may be explained by the surgical resection of the RSB in these studies, potentially accounting for the observed hyperactivity. Burkitt et al. found that postoperative colonic pressures were low compared to controls [35]. A further low‐resolution study also showed that early oral food challenges postoperatively could stimulate colonic motility in the immediate postoperative period (day one) following colorectal surgery. This implies that food after colorectal surgery may increase colonic motility, with the caveat that low‐resolution data of this nature have limited reliability [37, 51].

### 3.2 LARS

Four studies investigated low anterior resection syndrome (LARS); three of which were HRM studies (Table 3). LARS describes the disorder of bowel function after distal colorectal resection, leading to a significant impact on the patient's quality of life [57]. The definition of LARS is broad but aims to capture a broad array of symptoms including fecal incontinence, urgency, and difficulty evacuating [57]. These HRM studies have helped clarify the clinical significance of the RSB. Keane et al. evaluated patients with HRM following previous distal colorectal resection and compared them to controls. The symptoms of these patients were assessed using the LARS Score [58]. They demonstrated altered postprandial responses, with fewer antegrade and retrograde propagating contractions compared to controls, and a markedly reduced proportion of CMPs following meals (26% vs. 58%) [2]. Following rectosigmoid resection, Vather et al. demonstrated that there is still a meal response and pressure wave propagation across an anastomosis [3]. These findings demonstrate altered distal colonic motility in those following colorectal resection, supporting the notion of an RSB. This colonic activity has clear importance in maintaining continence, as seen in these patients who have reduced antegrade and retrograde contractions and a higher symptom burden than controls [2].

**TABLE 3:** Studies investigating low anterior resection syndrome (LARS).

Author	Technique	Participants	Main findings	Limitations
Bassotti—2005	Water perfused catheter with 12 cm spaced sensors in mid‐right colon	Case report	Reduction of contractile segmental activity with more high‐amplitude propagated contractions compared to healthy subjects	Case report
Vather—2016	36‐channel HR fiber‐optic manometry, 1 cm sensor spacing, in distal colon	15 patients with distal colorectal resections > 12 months ago and 12 controls	Dominant frequencies in distal colon were similar (2–3 cpm). Motility and meal responses are restored after distal colorectal resection	Recordings made in a prepared colon
Keane—2021	36‐channel HR fiber‐optic manometry, 1 cm sensor spacing, in distal colon	23 patients (11 no‐LARS; 12 LARS) and nine controls	Rectosigmoid resection alters the meal response following anterior resection. LARS patients had fewer post‐prandial antegrade propagating contractions than controls and fewer retrograde propagating contractions both pre‐ and post‐prandially	Studies performed on ‘prepared colons’
Asnong—2022	Solid state catheter with 40 pressure sensors, 2.5 cm apart in caecum	18 patients (9 no/minor LARS, 9 major LARS)	Increased cyclic short antegrade motor patterns and a reduction in HAPS originating proximally	Small sample size. 2.5 cm spacing vs. 1 cm

In contrast, Asnong et al. used a solid‐state 2.5 cm solid‐state HRM catheter and found increased cyclic short antegrade motor patterns with a reduction in HAPS, originating in the proximal colon and terminating in the mid‐colon [43]. These divergent results may be due to patient population differences and/or technical differences in low vs. high resolution catheters as described by Dinning et al. [51].

These findings offer insights into the mechanisms of LARS. Even in a low‐resolution case report, Bassotti et al. comment on the potential effect of surgical resection of the RSB and its potential effect on altering transit into the rectum, contributing to frequency and urgency [42]. Long‐term restoration of normal colonic motor patterns and meal responses, including the RSB, has been demonstrated after distal colorectal resections; however, the propagation extent of cyclical patterns has been found to be shorter [3].

### 3.3 Acute Colonic Pseudo‐Obstruction (ACPO)

In a single high‐resolution case study using a fiber‐optic catheter, Wells et al. revealed disordered, non‐propagating hyperactive motility in the distal colon, which likely acts as a functional pseudo‐obstruction, causing secondary proximal colonic dilatation [11]. This contradicts the common assumption of general colonic hypoactivity in ACPO but supports the concept of distal colonic spasmodic activity [59]. These data are limited by the fact that this is a case report with no baseline recordings prior to decompression or in a non‐obstructed period. However, these novel findings present a uniquely detailed physiological insight into the mechanism of this disease, and data may help direct future research in larger cohorts to validate these findings and evaluate possible therapeutic interventions.

### 3.4 Diverticulosis

Nine studies evaluated diverticulosis with manometry, although only one of these was an HRM study (Table S1). The single HRM study by Jaung et al. demonstrated no evidence for increased manometric pressure or increased colonic activity in patients with diverticular disease compared to healthy controls; therefore suggesting that increased intracolonic pressures are potentially not the primary mechanism for diverticulosis formation [30]. They further demonstrated that patients with diverticulosis exhibited reduced post‐meal increase in retrograde propagating contractions compared with non‐age‐matched controls. The findings suggest potential alterations in colonic motility, specifically a decrease in the distance of propagation, which may be linked to other factors, such as changes in pacemaker cells (interstitial cells of Cajal), decreased colonic compliance, or altered collagen structures in the colonic wall. However, a limitation of this study by Jaung et al. was that patients and controls were not age‐matched, and these findings may therefore partly reflect potential age‐related changes in colonic motility [60]. The study highlights the need for further research into other contributing factors that may lead to diverticulosis.

By contrast, low‐resolution studies, such as those of Bassotti et al., showed that patients with diverticular disease displayed significantly increased motility in the affected segments (descending and sigmoid colon) compared to controls [22]. Patients with symptomatic, uncomplicated colonic diverticulosis were found to often display increased duration of rhythmic, low‐frequency, contractile activity (2–3 cpm), particularly in segments with diverticula [23]. While some studies showed higher motor activity in diverticulosis patients, especially those with constipation, weak evidence was found overall for consistently abnormal intraluminal pressure in the sigmoid colon under basal (resting) conditions. Sigmoid colectomy has been suggested for symptomatic diverticular disease, and a low‐resolution water‐perfused manometry study showed a significantly lower intraluminal pressure postoperatively, which continued out to 3–5 years [24].

Despite the fact that these data demonstrate differences in those with diverticulosis versus healthy controls, the low‐resolution studies are less reliable than the HRM data and demonstrate the value of the new HR techniques [51]. Taken together, the low and high resolution findings can be seen as partly complementary as these differences may reflect not only methodological differences but also heterogeneity in patient populations (symptomatic vs. asymptomatic, age‐related changes, or co‐existing constipation). The overall evidence suggests that diverticulosis is unlikely to be explained solely by high intracolonic pressure; instead, altered motility, compliance, and wall structure likely interact in its pathophysiology.

### 3.5 Fecal Incontinence

Five studies investigated FI and the efficacy of SNS in its treatment, of which two were high‐resolution (Table S2). Lin et al. demonstrated that patients with FI have a generally suppressed RSB function, with fewer retrograde propagating contractions than healthy controls after meals [10]. These patients were then treated with sacral nerve stimulation (SNS), which, compared to baseline, showed a significant increase in the retrograde propagating contractions in the distal colon, although not coming close to the motility levels seen in healthy controls [10]. Furthermore, the HRM study by Patton et al. also found a significant increase in the frequency of retrograde propagating sequences in those with true SNS compared to sham stimulation. Compared with sham stimulation, SNS had no effect on the frequency of antegrade PSs or high‐amplitude PSs [34].

A low resolution study by Herbst et al. found that HAPS were seen to be similar in FI compared to subjects with normal bowel control, with the key difference in urge incontinence patients appearing to be an inability of the RSB to oppose these high rectal pressures adequately [33].

As mentioned, SNS modulates colonic motility in patients with fecal urge incontinence and has been shown to enhance retrograde cyclic activity in the left colon [10, 34]. These changes to retrograde propagating sequences and improvement of the function of the RSB suggest a highly plausible role for colonic motility in the pathophysiology of fecal incontinence and a mechanism for SNS's therapeutic action [10]. These findings correlate with symptomatic improvement in FI; this may also be true for LARS when treated with SNS; however, no studies were found on this latter therapy indicating a need for further investigation.

### 3.6 Defunctioned Distal Colon

Pucciani et al. demonstrated in a low‐resolution study of colostomy using water‐perfused manometry that the motility of the distal defunctioned colon is preserved in patients with a temporary transverse loop colostomy [21]. Both proximal and distal segments showed a significant increase in motor activity after a standard meal, although the distal colon consistently exhibited significantly less motor activity than the proximal colon. Given the low‐resolution nature of this study, the findings must be cautiously interpreted.

### 3.7 Inflammatory Bowel Disease

Two studies were identified in ulcerative colitis, both of which used low‐resolution manometry. These two studies found that colonic motor activity is frequently abnormal in patients with UC, even when inflammation is mild or moderate. Those with active disease were found to have rapid transit and increased propulsive activity [61]. Both HAPS and low‐amplitude propagated sequences (LAPS) were significantly increased in patients with active UC. This increased propulsive activity is hypothesized to be due to increased secretory function and the inflammatory mucosal process, potentially facilitating forward movement due to decreased segmental contractions [41].

### 3.8 Medication

Various surgically‐relevant pharmacological agents and interventions can significantly alter colonic motility.

#### 3.8.1 5‐HT3 Antagonists (Ondansetron, Tropisetron)

Ondansetron reduces the post‐meal colonic hypertonic response in carcinoid diarrhea and increases post‐prandial phasic activity. Thus, it potentially has a constipating effect by modulating the RSB [48]. Tropisetron significantly increases the colonic motility index in the early postoperative period; however, it was observed to impair the gastrocolonic response after meal administration [46].

#### 3.8.2 Cisapride

A low‐resolution study demonstrated a possible differential effect on the colon proximal and distal to a stapled/handsewn left‐sided colonic anastomosis, and significantly increased indices of motility in the early postoperative period although these are unlikely to be clinically significant [47].

#### 3.8.3 Opioids and Cholinergics

Two low‐resolution studies from the 1960s showed that morphine immediately causes a rise in basal intraluminal pressure and a succession of high‐pressure waves, and facilitates localized high intrasigmoid pressures in diverticulosis [27, 44]. Pethidine almost abolishes the generation of high‐pressure waves in the sigmoid, in both healthy and diverticulosis states [44]. Probanthine works as an anti‐cholinergic and counteracts morphine's adverse effects by paralyzing the bowel and, as such, may prevent high‐pressure generation [44].

#### 3.8.4 Neostigmine

This drug significantly increased the colonic motility index (attributed to higher frequency and greater amplitude of contractions) in postoperative patients and healthy volunteers [44, 45]. Corsetti et al. found using HRM that prostigmine causes pan‐colonic pressurizations, although this was in a cohort of those with chronic constipation [49]. These data suggest its effectiveness in stimulating colonic motility and tone in the early postoperative period. It is also noted as an effective treatment for colonic decompression in ACPO [11].

## 4 Discussion

**FIGURE 1:**
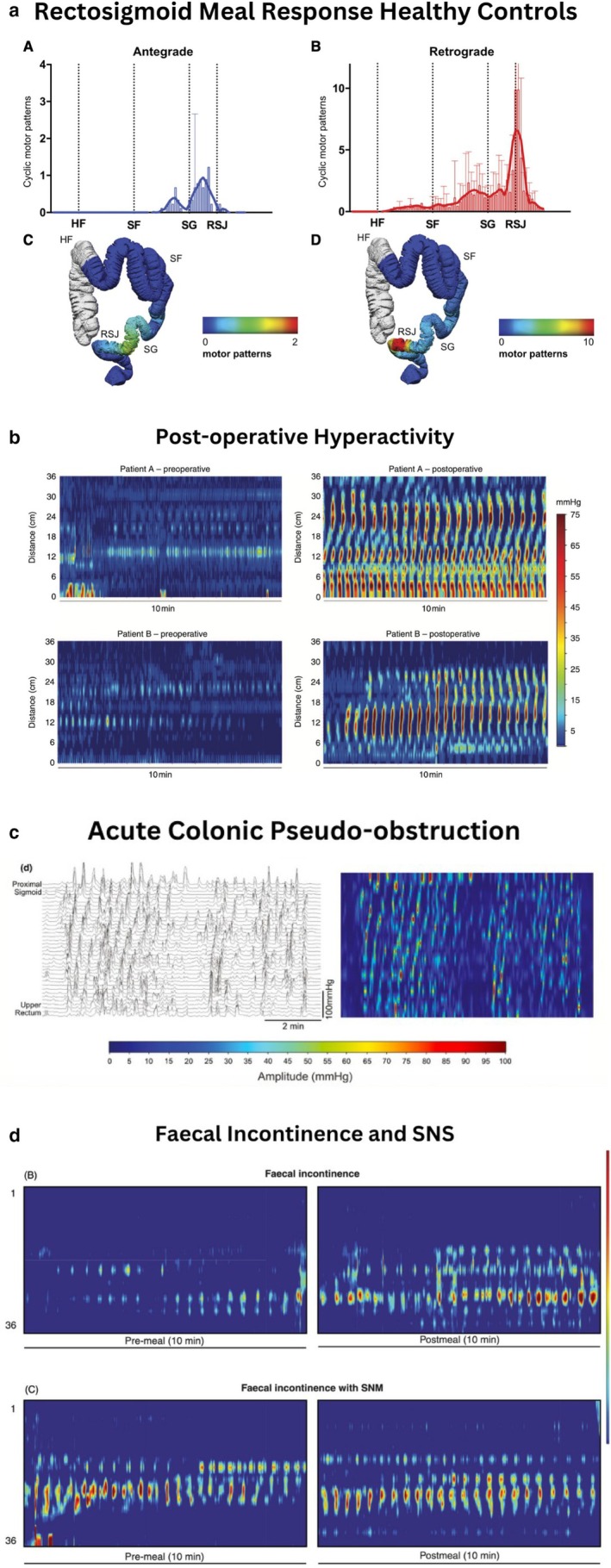
Rectosigmoid brake across health and disease. Representative high‐resolution colonic manometry (HRM) plots illustrating key motor patterns in the distal colon under physiological and pathological conditions. (a) Healthy controls demonstrate a normal rectosigmoid brake, with meal‐induced augmentation of retrograde cyclic motor patterns (2–6 cpm) localized to the sigmoid colon (Adapted with permission from Lin et al. [9] (license #1666440‐1)). (b) In the early postoperative period, patients with ileus exhibit disorganized or hyperactive cyclic activity lacking coordinated propagation. Postoperative colonic hyperactivity (Adapted with permission from Vather et al. [4] (license #6145191284690)). (c) In acute colonic pseudo‐obstruction, uncoordinated high‐amplitude non‐propagating contractions are seen, reflecting failure of organized distal colonic motility (Adapted from Wells et al. [11]. This article is available under the terms of the Creative Commons Attribution License. PMCID: PMC8261480). (d) Patients with fecal incontinence show an attenuated rectosigmoid brake, with reduced frequency and amplitude (mmHg) of retrograde propagating contractions. SNS partially restores this function, increasing retrograde cyclic activity and re‐establishing post‐meal responsiveness (Adapted from Lin et al. [10]. This article is available under the terms of the Creative Commons Attribution License. PMCID: PMC10084032).

This review examines the role of RSB activity in surgical disorders of clinical significance. Colonic manometry, and particularly HRM, consistently demonstrates reproducible motor patterns consistent with a braking mechanism in the rectosigmoid region (Figure 1) [2, 4, 7, 8]. Disruption of RSB activity, as found in many high‐resolution studies, indicates this critical motility pattern to be a potential common and previously under‐appreciated pathway across distinct surgical syndromes, linking ileus, low anterior resection syndrome symptoms, including urgency, incontinence, and acute colonic pseudo‐obstruction through altered patterns of cyclic motor activity, sometimes with concomitant dysregulated HAPS. However, interpretation is limited by the predominance of low‐resolution manometry, with only 10 of 34 studies employing HRM, and with HRM data largely confined to disparate conditions, of which only two have been examined more than once. This reflects a weak and fragmented high‐resolution evidence base and underscores the need for further HRM studies in comparable clinical populations. This convergence of data, consolidated in the current systematic review, suggests that the RSB is not merely a regional motor feature but rather a central coordinator of colorectal function, with direct clinical relevance across multiple domains. Importantly, its responsiveness to neuromodulation (e.g., SNS) highlights the possibility of targeted therapies to restore physiological motility rather than treating symptoms in isolation of fundamental underlying mechanisms. Notably, there is a substantial temporal gap between early, widely cited studies and more recent investigations, reflecting the historical technical limitations of colonic motility assessment. Advances in high‐resolution and non‐invasive techniques have therefore been central to recent progress in understanding rectosigmoid motor physiology and its clinical relevance.

Critical recent technical advances include the development of HRM catheters together with validated ambulatory HRM systems, enabling prolonged physiological recordings outside the clinical setting and providing more representative data on day‐to‐day colonic activity [3, 62]. These techniques also facilitate appropriate interpretation of frequency and the directionality of propagating sequences, which have been crucial to defining RSB physiology and pathophysiology as reported here [8, 51]. Despite these improvements, direct correlation of pressure events with wall motion and luminal transit remains difficult, and therefore concomitant adjacent imaging studies remain relevant [38, 63]. While such studies were outside the current scope, the potential for multi‐modal convergence to fully define pathophysiology and clinicopathological correlation, beyond manometry alone, was demonstrated in a recent study by Chapman et al. using MRI in postoperative ileus [64]. This recent study, currently in abstract form, confirmed HRM data showing rectosigmoid hyperactivity as a plausible key contributing mechanism to delayed return of bowel function after right‐sided colonic resections, consistent with the current review's findings.

Clinical correlations identified in this systematic review demonstrate that preservation or disruption of the rectosigmoid segment has important consequences for colonic motility, postprandial responses, and longer‐term bowel function. Observations from high‐resolution manometry (HRM) studies support the rectosigmoid brake (RSB) as a mechanistic link between surgical intervention and downstream functional sequelae. Low anterior resection syndrome (LARS) is widely recognized as the principal determinant of quality of life following rectosigmoid surgery, and altered distal colonic motility represents one plausible contributor within this multifactorial condition [65, 66].

Further evidence for the clinical relevance of the RSB is provided by studies examining pharmacological and neuromodulatory interventions. Agents including 5‐HT_3_ antagonists, opiates, cholinergic drugs, and sacral nerve stimulation (SNS) have all been shown to modulate distal colonic motor activity. However, many of these studies pre‐date the formal description of the rectosigmoid brake or used relatively immature techniques, such as the early cineradiography studies of Painter et al. [44]. Consequently, their specific effects on rectosigmoid motor patterns often still remain unclear, highlighting the need for targeted investigation using high‐resolution techniques.

Although hyperactive distal cyclic motor patterns in the early postoperative setting may appear counterintuitive, this finding has been remarkably consistent across all patients studied using high‐resolution techniques in two independent cohorts [4, 40]. Moreover, similar patterns align with clinical observations of relatively delayed recovery following right‐sided resections compared with left‐sided resections or high anterior resections, where the rectosigmoid segment is removed, suggesting that these changes are unlikely to be solely attributable to opiate or anesthetic effects. Interestingly, a study by Frantzides et al. [67], not included in this review, assessed the effects of morphine using serosal electrodes rather than manometry, demonstrating stimulation of colonic spike‐burst electrical activity, particularly in the sigmoid colon. Further studies are therefore required to disentangle the relative contributions of neurohormonal versus pharmacological mechanisms in postoperative bowel hyperactivity, and to define its significance as a therapeutic target for enhanced recovery.

In FI, these findings support that manometric evidence of RSB dysfunction may underpin impaired continence control and that restoration of this activity represents a plausible mechanism of action for SNS. While traditional explanations of SNS have emphasized sensory or afferent neural pathways, emerging motility data now provide persuasive evidence that modulation of distal colonic motor patterns contributes meaningfully to its therapeutic effect. However, most studies to date include relatively small patient numbers, reflecting the invasive nature of current measurement techniques. Consequently, conclusions regarding optimal patient selection and translation of these physiological findings into clinical practice remain preliminary. Given the incomplete response rates to current therapies, their invasiveness, and the potential for physiological biomarkers such as the RSB to guide treatment selection or stimulation protocols, further prospective translational research should be prioritized [8].

This is the first systematic review to specifically evaluate the clinical significance of the RSB. The strengths of our review include a comprehensive search strategy, clear inclusion criteria targeting surgically relevant populations, and a structured synthesis of results across multiple surgically relevant conditions. However, our findings are limited by the quality and heterogeneity of the included studies, as well as the inability to perform a meta‐analysis due to variable outcome reporting. In particular, we find the available literature to be limited, with most studies being small, heterogeneous, and inconsistent in methodology. Variability in catheter resolution, placement, recording duration, differing study protocols, and definitions of motor patterns make comparisons difficult. Many of the studies only utilized low‐resolution manometry techniques, affecting the quality of the data and therefore the interpretations. In addition, many studies were observational or exploratory, with limited statistical power. Lastly, few studies included healthy control groups, and normative data on rectosigmoid activity remains scarce. Importantly, while rectosigmoid motor patterns are often visually described, objective and reproducible criteria for defining the RSB are also inconsistently applied across studies. There is also a need to distinguish physiologic variations from clinically significant dysfunction. Studies need to unify terminology, analytic criteria, and reporting standards across manometric research, for example, as proposed by Corsetti et al. [68].

While a broad initial evidence base could be synthesized, to advance the field further, there is a need for further prospective, standardized studies using high‐resolution techniques in both healthy volunteers and surgical patients. Normative reference ranges should be established, and the relationship between RSB activity and clinical outcomes should be further elucidated. However, research momentum has been impaired by the invasive nature of the technologies, which have typically required bowel preparation, colonoscopy, and the insertion and maintenance of manometry catheters, sometimes for days, which is relatively invasive.

Recent advances in non‐invasive surface mapping techniques offer a promising opportunity to overcome many of the limitations that have historically constrained investigation with traditional manometric techniques. New non‐invasive approaches to assess gut function, including the RSB, have been developed. In addition to imaging as mentioned above [63], the development of non‐invasive body mapping and its validation may provide an optimal methodology to achieve larger study sizes in diverse disease states [69, 70]. Recent gastric studies of this type have demonstrated how large cohorts can be assessed with standardized reference ranges and symptom correlations: techniques that may now be readily adapted to the colon [71, 72]. There is also an opportunity to validate motility‐based biomarkers as tools for preoperative risk stratification or therapeutic response. Translational studies should, for example, evaluate whether RSB activity predicts surgical outcomes, guides neuromodulation therapies, or stratifies LARS patients for interventions. Ultimately, validating the RSB as both a biomarker and a therapeutic target could reshape approaches to postoperative bowel dysfunction.

Additionally, emerging evidence suggests that the RSB is modulated not only by luminal and reflexive mechanisms, but also by higher‐order neural inputs via the gut‐brain axis. Psychological factors such as stress and anxiety may alter colonic motility patterns through autonomic dysregulation, potentially exacerbating symptoms in functional bowel disorders. In surgical patients, for example, data have shown that preoperative anxiety and pain may influence colonic motor responses, including cyclic activity in the rectosigmoid region [73]. Future studies should therefore investigate the role of psychophysiological modulation in RSB function and its interaction with surgical stress and recovery.

If pre‐ and postoperative colonic motility can be monitored non‐invasively, the RSB may serve as a clinically valuable biomarker that predicts postoperative recovery and treatment response, an avenue not yet realized in clinical practice. Recent technological advances have made this increasingly feasible. High‐resolution body surface mapping techniques now allow assessment of gastrointestinal motor patterns without the need for intraluminal catheters. Pioneering work by Erickson et al. and Seo et al. has demonstrated the capacity of high‐density surface recordings to characterize spatiotemporal colonic activity, while novel flexible electrode systems have been developed to enable scalable and patient‐tolerant recording platforms [69, 70, 74]. These innovations highlight a translational frontier where physiological signatures such as the RSB could be tracked longitudinally, before and after surgery, or in response to neuromodulatory interventions, ultimately establishing a new class of non‐invasive biomarkers for surgical recovery and gut function.

## 5 Conclusion

This review finds that the RSB represents a physiologically relevant and clinically important mechanism of colonic motility with broad implications for surgical diseases and outcomes. Disruption of this critical motor pattern is associated with several postoperative and surgical syndromes, including POI, LARS, FI, and ACPO. As manometric and non‐invasive mapping technologies evolve, incorporating RSB assessment into clinical practice may help guide therapeutic decision‐making and improve functional recovery. Future work should focus on translating the clear potential of the RSB to impact practice, including by standardizing definitions, refining and advancing non‐invasive measurement techniques, and validating the RSB as a therapeutic target.

## Author Contributions

J.A.P.: study design, data extraction, data analysis, primary drafting of manuscript. All authors: study design and oversight, data interpretation and critical manuscript review. All authors are qualified for authorship, have approved the final version of this manuscript, and agree to the accountability of its accuracy and integrity.

## Funding

This work was supported by the Health Research Council of New Zealand.

## Conflicts of Interest

G.O.G. has grants and patents in gastrointestinal motility, and is a founding member of Alimetry and The Insides Company. I.P.B. is a founding member of The Insides Company. All other authors have no competing interests.

## Supporting information


**Figure S1:** nmo70288‐sup‐0001‐DataS1.docx PRISMA diagram.
**Table S1:** Studies Investigating Diverticulosis.
**Table S2:** Studies investigating fecal incontinence.

## Data Availability

No new data were generated or analyzed in this study. Data sharing is not applicable to this article.
