# Supplementary material for: The Clinical Relevance of the Rectosigmoid Brake in Surgical Disorders and Therapies: A Systematic Review of Colonic Manometry Studies
Source: Neurogastroenterol Motil. 2026 Mar 19;38(3):e70288. doi: 10.1111/nmo.70288 (PMC13002558; doi:10.1111/nmo.70288)
Supplement: Supplementary file 1 — Figure S1: nmo70288‐sup‐0001‐DataS1.docx PRISMA diagram. Table S1: Studies Investigating Diverticulosis. Table S2: Studies investigating fecal incontinence. [file NMO-38-e70288-s001.docx]

**Appendix**

***Supplementary Figure 1:*** *PRISMA Diagram*

**

***Supplementary Table 1:*** Studies Investigating Diverticulosis

| **Author** | **Technique** | **Participants** | **Main Findings** | **Limitations** |
| --- | --- | --- | --- | --- |
| Painter – 1965 | Three water perfused catheter tips spaced 7.5cm apart, above ‘rectosigmoid’. | 32 controls with 28 patients. | Under resting conditions there is no difference in colonic pressures between healthy controls and patients. Morphine cause a rise in basal intra-luminal pressure followed by a series of high pressure waves. | Low-resolution catheter and cineradiography. |
| Eastwood – 1980 | Pressure transducer catheter, 5cm spacing (15, 20, 25cm from anal verge). | 19 patients. | No correlation between colonic motility index and intestinal transit. | No control group. Low resolution catheter. |
| Smith – 1981 | Pressure transducer catheter in ‘rectosigmoid’. | 24 patients. | Coarse bran versus fine bran significantly increased the colonic motility index. | Manometry catheter not described. No control group. |
| Cortesini – 1989 | Two single point water perfused catheters in descending and sigmoid colon. | 20 controls, 55 patients. | Patients with symptomatic complicated diverticular disease had a significantly higher motility index. Anterior resection significantly reduces intraluminal pressures. | Low resolution. |
| Katschinski – 1990 | Single point water perfusion catheter in rectosigmoid. | 41 patients with IBS, 15 with diverticulosis, 13 controls. | No significant pressure differences at baseline or post-prandially in any group. | Low resolution, single point manometry. |
| Viebig – 1994 | Water perfused catheter with two channels 10cm apart in ‘rectosigmoid’. | 19 ‘healthy’,  70 constipation,  21 IBS,  19 diarrhoea,  13 localized diverticulosis,  31 generalized diverticulosis,  23 diverticulitis | No significant pressure differences. Hypermotility in diverticular disease is probably due to the presence of constipation. | Heterogenous study pool. Low resolution. No true controls. |
| Basotti – 2001 | Water perfused, 8 channel catheter with 12cm spacing in at least proximal transverse colon. | 10 patients with symptomatic uncomplicated diverticular disease, 16 controls. | Patients with diverticular disease had significantly increased motility in the descending and sigmoid colon compared to control subjects with a significant increase in HAPS averaging 10.3 per subject per day compared to 5.5 in controls. | Baseline characteristic not evenly distributed. Low resolution. |
| Basotti – 2005 | Water perfused, 8 channel catheter with 12cm spacing in at least proximal transverse colon. | 12 patients with symptomatic uncomplicated diverticular disease. | Patients displayed increased duration of rhythmic, low-frequency, contractile activity, particularly in the segments bearing diverticula (increase in 2-3cpm) | No controls. 8 women, 4 men. Low resolution. |
| Jaung – 2021 | Fiber optic HRM with 72-sensors at 1 cm intervals positioned at hepatic flexure. A shorter 36-sensor  catheter was used in only one case. | 9 controls, 9 patients with established asymptomatic diverticulosis. | No evidence for increased manometric pressures or increased colonic activity in patients with diverticular disease compared to healthy controls. Diverticulosis patients also exhibited a reduced post-meal increase in retrograde propagating contractions | Unmatched baseline characteristics. |

***Supplementary Table 2:*** Studies Investigating Faecal Incontinence

| **Author** | **Technique** | **Participants** | **Main Findings** | **Limitations** |
| --- | --- | --- | --- | --- |
| Herbst – 1997 | 5-channel 72cm water perfused catheter in transverse colon. | 6 patients, 6 controls. | Urge incontinence, urge to defaecate, and normal defaecation have identical HAPS. The difference in urge incontinence patients appears to be an inability of the anal sphincter to adequately oppose these high rectal pressures. | Small sample size. Only women. |
| Gallas – 2009 | 12-lumen water perfused catheter with 10cm spacing in sigmoid colon. | 12 controls. | Short-term non-invasive magnetic stimulation over the sacrum delayed the onset of bisacodyl-induced HAPS but did not alter the frequency, amplitude, or duration of HAPS compared to sham stimulation. These findings suggest that SNS may exert its therapeutic effect in FI by inhibiting propulsive colonic motility. | Small sample size. Low resolution. |
| Rodger – 2010 | Water perfused, 180-cm multilumen manometry in descending / sigmoid colon. | 15 patients, 5 controls. | Patients with urge faecal incontinence demonstrated increased colonic motor activity, showing significantly more LAPS in the sigmoid and descending colon and more HAPS in the sigmoid colon during fasting. This suggests abnormal proximal colonic motility at rest. | Only women. Compared to a control group that was not described. |
| Patton – 2013 | 90-channel HRM solid-state, fiber-optic catheter with 1cm spacing in caecum. | 11 patients. | If a significant outlier was removed from the data, true stimulation in the  remaining ten patients resulted in a significant increase  in the frequency of retrograde PSs in comparison with sham stimulation. | 10 women, 1 male. |
| Lin – 2022 | 36-channel HRM fiber-optic catheter with 1cm spacing in descending / sigmoid colon | 15 patients, 10 controls. | Patients with FI exhibit an attenuated RSB, which is improved by SNS. SNS significantly increased total and retrograde propagating contractions in the fasting state, while antegrade contractions remained unchanged. This indicates that SNM helps partially restore the impaired RSB and meal response seen in FI. | Small sample size. Different bowel prep used in control and patient groups. |
